# The POU-HD TFs impede the replication efficiency of several human papillomavirus genomes

**DOI:** 10.1186/s12985-024-02334-w

**Published:** 2024-03-05

**Authors:** Martin Kala, Sofiya Babok, Nika Mikhailava, Marko Piirsoo, Alla Piirsoo

**Affiliations:** Institute of Technology, University of Tartu, Tartu, Estonia

**Keywords:** Human papillomavirus, POU-homeodomain transcription factors, Replication, Transcription

## Abstract

Human papillomavirus (HPV) is a double-stranded DNA virus that infects cutaneous and mucosal epithelial cells. HPV replication initiates at the origin (ori), located within a noncoding region near the major early promoter. Only two viral proteins, E1 and E2, are essential for replication, with the host cell contributing other necessary factors. However, the role of host cell proteins in regulating HPV replication remains poorly understood. While several binding sites for cellular transcription factors (TFs), such as POU-HD proteins, have been mapped in the regulatory region, their functional importance is unclear. Some POU-HD TFs have been shown to influence replication in a system where E1 and E2 are provided exogenously. In this study, we investigated the impact of several POU-HD TFs on the replication of the HPV5, HPV11, and HPV18 genomes in U2OS cells and human primary keratinocytes. We demonstrated that OCT1, OCT6, BRN5A, and SKN1A are expressed in HPV host cells and that their overexpression inhibits HPV genome replication, whereas knocking down *OCT1* had a positive effect. Using the replication-deficient HPV18-E1^−^ genome, we demonstrated that OCT1-mediated inhibition of HPV replication involves modulation of HPV early promoters controlling E1 and E2 expression. Moreover, using Oct6 mutants deficient either in DNA binding or transcriptional regulation, we showed that the inhibition of HPV18 replication is solely dependent on Oct6’s DNA binding activity. Our study highlights the complex regulatory roles of POU-HD factors in the HPV replication.

**Supplementary Information:**

The online version contains supplementary material available at 10.1186/s12985-024-02334-w.

## Introduction

POU-homeodomain (POU-HD) proteins constitute a class of transcription factors (TFs) in eukaryotes, with six subclasses identified based on sequence homology in the DNA binding domain (DBD). Mammals harbour a total of 16 genes belonging to this class, which exhibit diverse functions in ontogenesis, cellular homeostasis, and disease progression [1]. The expression patterns of POU-HD genes range from ubiquitous, exemplified by OCT1, to highly cell type-specific, such as PIT1 in the pituitary gland [2, 3].

The DBD of POU-HD proteins comprises two subdomains: the homeodomain and the POU-specific domain. The founding members Oct1 and Oct2 bind to an A/T-rich consensus ATGCAAAT site. However, the bipartite structure of the DBD, coupled with the ability of POU-HD proteins to form homodimers and heterodimers, facilitates high-affinity binding to diverse DNA sequences. The outcome of POU-HD binding, whether activation or repression of transcription, depends on the presence of cofactors and other binding sites near the POU-HD sites [1].

Previous research indicates that POU-HD proteins play a role in regulating DNA viral genome replication. For instance, OCT1, a ubiquitously expressed POU-HD protein, stimulates adenovirus replication by tethering the viral initiator protein pTP to the origin of replication (ori) [4, 5]. Conversely, OCT1 binding to SV40 ori inhibits DNA unwinding during replication initiation [6]. Ectopic expression of OCT6 has been shown to stimulate early transcription of gliotropic polyomavirus JC, indirectly promoting viral genome replication in glioblastoma cells, a phenomenon not observed with OCT1, OCT2, or PIT1 expression [7].

Papillomaviruses, a family of double-stranded DNA viruses, infect basal cells of stratified cutaneous and mucosal epithelia. Infections by these viruses can be asymptomatic or cause various dysplasias, ranging from benign warts to neoplastic cancers. Human papillomavirus (HPV) types are categorized into low- and high-risk types (HR and LR, respectively) based on their potency to induce dysplasia. While LR HPVs lead to benign lesions, HR types are associated with cancer development, including anal, cervical, vulvar, penile, and head-and-neck carcinomas [8].

The HPV genome undergoes replication within the nuclei of infected cells as an extrachromosomal episome. Comprising at least eight open reading frames (ORFs) coding for viral proteins and a noncoding region, the upstream regulatory region (URR), the viral genome relies on a short sequence element within the URR termed the ori for initiation. The essential components for HPV replication include ori as a cis element and E1 and E2 proteins as trans-acting factors. E1, a viral initiator protein, binds to ori and serves as a helicase during replication, while E2 acts as the primary viral transcriptional activator, facilitating E1 binding to the ori sequence. The number of E2 binding sites needed for functional ori sequences varies depending on the HPV type [9].

In addition to E1 and E2 binding sites, the URR contains numerous binding sites for host cell TFs, most of which have been predicted *in silico* [10]. However, the impact of these sites and their associated factors on viral genome replication remains largely unclear. The study of cellular TFs in HPV replication is complicated by their potential indirect effects, modulating the expression of viral E1 and E2 proteins rather than replication per se.

Previous research on other DNA viruses suggests that the presence of cellular TF binding sites in the ori region generally benefits replication efficiency. For example, AP1 sites in the polyomavirus ori stimulate T antigen-mediated unwinding of DNA, and the TFs ZBP-89 and SP-1 enhance the binding of the viral initiator to the ori in Epstein-Barr virus [11]. Additionally, the AP1 and NF-1 binding sites augment replication efficiency from the SV40 ori [12, 13].

A comprehensive study on the influence of TF binding sites on viral replication analysed HPV31 genome replication in the human squamous cell carcinoma cell line SCC13 [14]. Mutations in AP1 and SP1 sites within the HPV31 URR region decreased replication efficiency, whereas mutations in an OCT1 site did not significantly affect replication. This decrease was attributed to lower activity of the viral early promoter, resulting in reduced expression levels of E1 and E2.

Fragmentary knowledge exists regarding the impact of POU-HD TFs on HPV replication. Studies have shown that SKN1A stimulates replication of HPV16 ori in HEK293 cells when E1 and E2 are provided in trans [15]. Conversely, OCT1, BRN1, and OCT6 had a negative effect in the same experimental context. POU proteins specifically expressed in the skin, OCT6, and SKN1A, were found to activate transcription from the HPV1A early promoter, suggesting an indirect positive influence on HPV replication [16]. Similar results were observed for HPV16 and HPV18, where SKN1A activated the viral early promoter, and BRN3A acted as a positive regulator of HPV16 early transcription [17, 18].

This article investigates the impact of several POU-HD proteins on the replication of the supposed HR beta HPV5, LR alpha HPV11, and HR alpha HPV18 genomes in U2OS cells and human primary epithelial keratinocytes (HPEKs). The study reveals that OCT1, OCT6, BRN5, and SKN1A are expressed in HPV host cells and exert a negative effect on the replication of the studied HPV genomes in both cell types. Additionally, the negative effect of OCT6 on the replication of the HPV18 genome is shown to be dependent on the bipartite DBD of the protein but not the activation domain.

## Materials and methods

### Plasmids

The viral genomes HPV5, HPV5-Nluc, HPV11, HPV18, HPV18-Nluc, and HPV18-E1^−^ were previously described [19–22]. These genomes were generated as minicircle plasmids [23, 24]. Expression vectors for human OCT1 (POU2F1) and BRN3A (POU4F1) were obtained from Addgene (catalogue numbers 53,306 and 62,221, respectively). OCT6 (POU3F1) and BRN2 (POU3F2) were PCR-amplified from U2OS-derived genomic DNA. Synthetic codon-optimized DNAs encoding human BRN5 (POU6F1) and SKN1A (POU2F3) were acquired from Genewiz. All POU TF (TF) DNAs were subcloned and inserted into the pCDNA3.1 vector (Clontech). Expression constructs encoding mouse Oct6 and its mutants N197 and WF > CS were kindly provided by Prof. Dies Meijer (University of Edinburgh).

### Cell culture

Human osteosarcoma U2OS cells (ATCC no HTB-96) were cultured in Iscove’s Dulbecco’s medium (Biowest) supplemented with 10% foetal calf serum, and 1% penicillin-streptomycin (Sigma-Aldrich). U2OS cells were transfected using electroporation and the Gene Pulser XCell system (Bio-Rad Laboratories) at a single pulse with capacitance 975 µF and voltage 220 V. The following amounts of the viral genomes were used per 10^6^ cells: 1.5 µg of HPV5, 1 µg of HPV11 and 1 µg of HPV18. Amounts of human POU-HD expression vectors per 10^6^ cells varied depending on experiments and are indicated in the respective figures.

HPEKs (CELLnTEC) were grown in defined keratinocyte-SFM medium (DKSM) (Gibco) supplemented with bovine pituitary extract, EGF and 1% penicillin-streptomycin. HPEKs were transfected 2 days after splitting using approximately 70 ng of the HPV18-Nluc genome, 10 ng of the POU-HD expression vectors, 0.12 µl of Plus reagent and 0.15 µl of Lipofectamine 3000 transfection reagent (Invitrogen) per well of the 96-well plate. HPEK differentiation was induced using 1.5 mM CaCl_2_ in DKSM with a 3-fold reduction in supplements.

### DNA isolation and southern blot (SB)

For isolation of total DNA, U2OS cells were washed with PBS and incubated with a lysis solution (20 mM Tris-HCl, pH 8.0, 100 mM NaCl, 10 mM EDTA, and 0.2% SDS). Lysates were homogenized by forcing each sample through a 26G syringe and treated with proteinase K (0.2 µg/µl) at 56 °C overnight. Following the phenol-chloroform extraction, the precipitated DNA pellet was resuspended in TE buffer containing RNase A (25 µg/ml) and incubated at 37 °C for 2 h. The DNA samples were re-precipitated and resuspended in TE.

Prior SB, approximately 4 µg of total DNA was treated with the following FastDigest restriction enzymes (Thermo Fischer Scientific): DpnI to digest bacterially purified transfected plasmids and SacI, BglI, and HindIII to linearize HPV5, HPV18 and HPV11 genomes, respectively. DNA transfer and hybridization were performed as described previously [25]. Radioactively labelled HPV DNA was visualized using a Typhoon Biomolecular Imager and quantified by ImageQuant software (Amersham).

### Western blot (WB)

U2OS cells were transfected either with an empty vector or a plasmid encoding the OCT1 using electroporation. Cells were incubated for 2 days and lysed in RIPA buffer (50 mM Tris-HCl, pH 7.5, 2 mM EDTA, 0.1% SDS, 150 mM NaCl, 0.1% Triton X-100) supplemented with Laemmli sample buffer and 100 mM DTT. Lysates were homogenized using a 26G syringe, incubated at 100 ^o^C for 5 min and analysed using SDS-PAGE with subsequent transfer to PVDF membrane (Amersham). The membranes were incubated in 5% non-fat milk diluted in PBS containing 0.1% Tween (PBS-T). OCT1 protein was assessed using anti-OCT1 antibody (Santa Cruz Biotechnologies, cat no sc-8024, 1:1000) combined with goat-anti-mouse-HRP IgG secondary antibody (Invitrogen, 1:10000). GAPDH was assessed using anti-GAPDH-HRP antibody (Invitrogen, 1:8000). Oct6 proteins were detected using anti-Oct6 antibody (a kind gift from Prof. Dies Meijer (University of Edinburgh), 1:1000). The antibodies were diluted in PBS-T containing 2% non-fat milk. The signals were visualized using a SuperSignal West Dura Extended Duration Substrate (Pierce) and X-Ray film (Agfa).

### Electrophoretic mobility shift assay (EMSA)

U2OS cells were transfected with POU-HD TF-encoding constructs, incubated for 2 days, and then lysed in 10 cell pellet volumes in WCE buffer (20 mM HEPES-KOH at pH 7.9, 400 mM KCl, 1 mM EDTA, 10 mM DTT, 10% glycerol, and protease inhibitor cocktail). The cell suspension underwent four cycles of snap-freezing in liquid nitrogen and thawing on ice, followed by centrifugation at 14,000 rpm for 30 min at 4 °C to remove cellular debris. The lysates were aliquoted and stored at -80 °C prior to analysis. Equal amounts of extract were used in an EMSA using 10 fmole of a 32P end-labelled double-strand oligonucleotide probe. Probe and protein were incubated on ice for 30 min in 20 mM HEPES-KOH at pH 7.9, 1 mM EDTA, 1 mM EGTA, 150 mM NaCl, 1 µg of poly(dI-dC) and 4% Ficoll in a total volume of 20 µL. Protein-DNA complexes and free probe were separated on a 4% polyacrylamide gel in 0.25× TBE electrophoresis buffer at 4 °C. Gels were fixed in 10% methanol/10% acetic acid, dried, and exposed to a Typhoon Biomolecular Imager.

The following double-strand oligonucleotide probes were used (the sense strand is shown): an OCTA probe (GAGAGGAATTTGCATTTCCACCGACCTTCC), and a BRN5 probe (GAGAGGGCATAAATAATTTCCACCGACCTT).

### Luciferase assay

The luciferase assay was performed in triplicates on 96-well plates. After transfection, U2OS cells or HPEKs were incubated for the indicated periods of time and washed with PBS. HPEKs were lysed in Passive lysis buffer (Promega). Nano-luciferase (Nluc) activities were measured using the Nano-Glo Luciferase Assay System (Promega) and normalized to alkaline phosphatase values measured with CSDP Substrate (Thermo Fischer Scientific). For U2OS cells, Nluc activities were measured using the Live Cell Assay (Promega) and normalized to viable/proliferating cell numbers measured with the MTS assay (Abcam).

### Cell cycle analysis

U2OS cells were detached using 0.5% trypsin and washed with PBS. The cells were fixed by pipetting ice-cold 70% ethanol drop-wise onto the cell pellet and gentle mixing, followed by incubation at 4 °C for 1 h. The fixed cells were washed with 3 ml of PBS containing 0.1% BSA (Sigma-Aldrich) and centrifuged at 2000 rpm at 4 °C. After treatment with RNase A (50 mg/ml) for 5 min, 6 µl of propidium iodide was added to 300 µl of cell suspension. Flow cytometry was performed using an Attune CytPix Flow Cytometer (Invitrogen).

### RNA isolation and RT-PCR

Total RNA was extracted using the Direct-Zol RNA Miniprep Plus kit (Zymo Research). Approximately 5 µg of total RNA was treated with 1.5 µl of Turbo DNase (Invitrogen) at 37 ^o^C for 2 h, followed by inactivation at 75 °C for 10 min and precipitation with 7.5 M LiCl containing 50 mM EDTA. Complementary DNA (cDNA) was synthesized using approximately 1 µg of the purified total RNA and RevertAid cDNA synthesis kit (Thermo Fisher Scientific). RT-PCR was performed using 5xHOT FIREPol Blend Master Mix with 12.5 mM MgCl_2_ (Solis Biodyne). Quantitative RT-PCR (qPCR) was conducted in triplicates using 5xHOT FIREPol EvaGreen qPCR Mix with ROX and LightCycle 480 Real-Time PCR System (Roche). Data were analysed using the comparative threshold cycle (ΔCt) method, and expression levels of the analysed transcripts were normalized to *GAPDH* mRNA expression levels. The primers used in the present study are listed in Supplementary Table S1.

### RNA interference

Two sets of *OCT1*-specific siRNAs and off-target scrambled control siRNAs were purchased from Santa-Cruz Biotechnology or Dharmacon. U2OS cells were transfected with 25 nM siRNAs using RNAiMAX transfection reagent (Invitrogen). The next day, the cells were detached using trypsin-EDTA and then transfected with the minicircle viral genomes by electroporation. The efficiency of the *OCT1* RNAi was analysed using qPCR with two independent pairs of oligonucleotides.

### Statistical analysis

P values were calculated using a two-tailed t test with equal variances assumption in Excel software.

## Results

### Expression pattern of POU TFs in HPEKs and U2OS cells

**Fig. 1:**
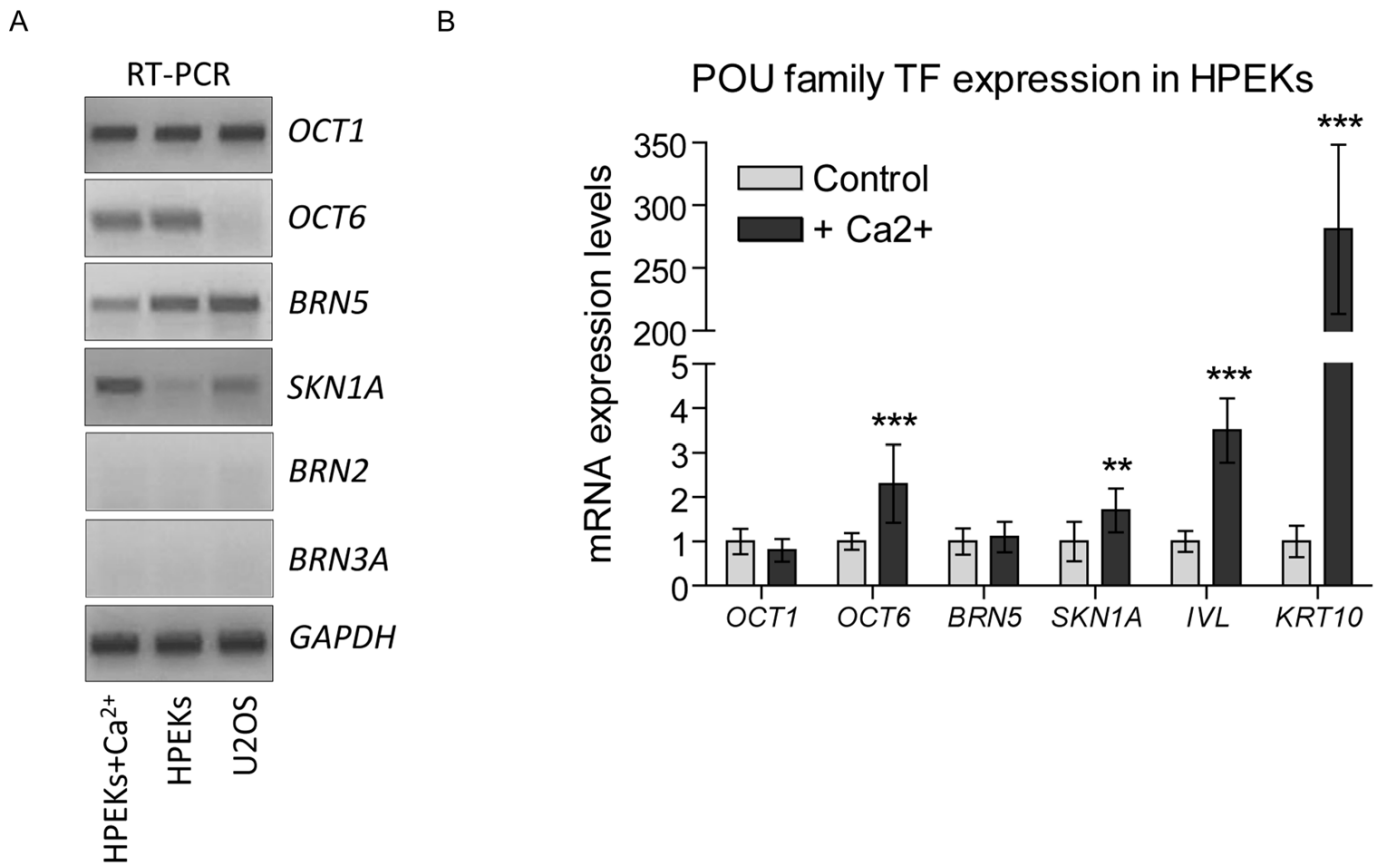
Expression of POU Family TFs in HPV Host Cells (**A**, **B**) HPEKs were treated with 1.5 mM CaCl_2_ for 2 or 3 days. Total RNA was isolated, treated with Turbo DNase, and subjected to cDNA synthesis. Expression patterns of the indicated POU TFs were analysed in U2OS cells and naïve and differentiation-committed HPEKs using RT-PCR (**A**) or qPCR (**B**). Gene expression levels were normalized to *GAPDH* mRNA levels, set as 1 in the untreated HPEKs, and data from other samples were calculated relative to 1; ** - *p* < 0.01, *** - *p* < 0.001, *n* = 3

To investigate the potential interference of POU TFs with HPV DNA, we assessed the expression of the family members *OCT1*, *OCT6*, *BRN2*, *BRN3A*, *BRN5*, and *SKN1A* in HPEKs and U2OS cells employing RT-PCR with two independent oligonucleotide pairs for each gene (Fig. 1A). HPEKs and U2OS cells were chosen as model cells capable of supporting HPV replication. Specificity of the oligonucleotides was confirmed using the respective expression constructs. Our analysis demonstrated the expression of *OCT1*, *OCT6*, *BRN5*, and *SKN1A* in HPEKs, which are natural host cells for HPV (Fig. 1A). In U2OS cells, the expression of the same genes, excluding *OCT6*, was detected. However, *BRN2* and *BRN3A* expression was undetectable in both cell types.

Considering the involvement of POU domain TFs in the regulation of epidermal keratinocyte gene expression, we explored the impact of Ca^2+^-induced differentiation on POU TF expression in three independent HPEKs batches using qPCR (Fig. 1B). HPEK were treated with Ca^2+^ for 2 or 3 days. We observed increased expression levels of *KRT10* and *IVL*, confirming HPEK commitment to differentiation. The expression levels of *BRN5* and *OCT1* remained comparable to those in undifferentiated HPEKs. However, *OCT6* and *SKN1A* mRNA expression increased by 2.3- and 1.7-fold, respectively, in response to Ca^2+^. Notably, Ct values for *BRN2* and *BRN3A* were higher than 37, indicating negligible expression (data not shown).

In summary, our data suggest that the TFs *OCT1*, *OCT6*, *BRN5*, and *SKN1A* may interact with HPV DNA, influencing its replication or transcription in infected human cells. The observed expression changes during HPEK differentiation further imply a dynamic role of these POU TFs in the context of HPV infection.

### Overexpression of OCT1 inhibits transient replication of HPV5 and HPV18 genomes

**Fig. 2:**
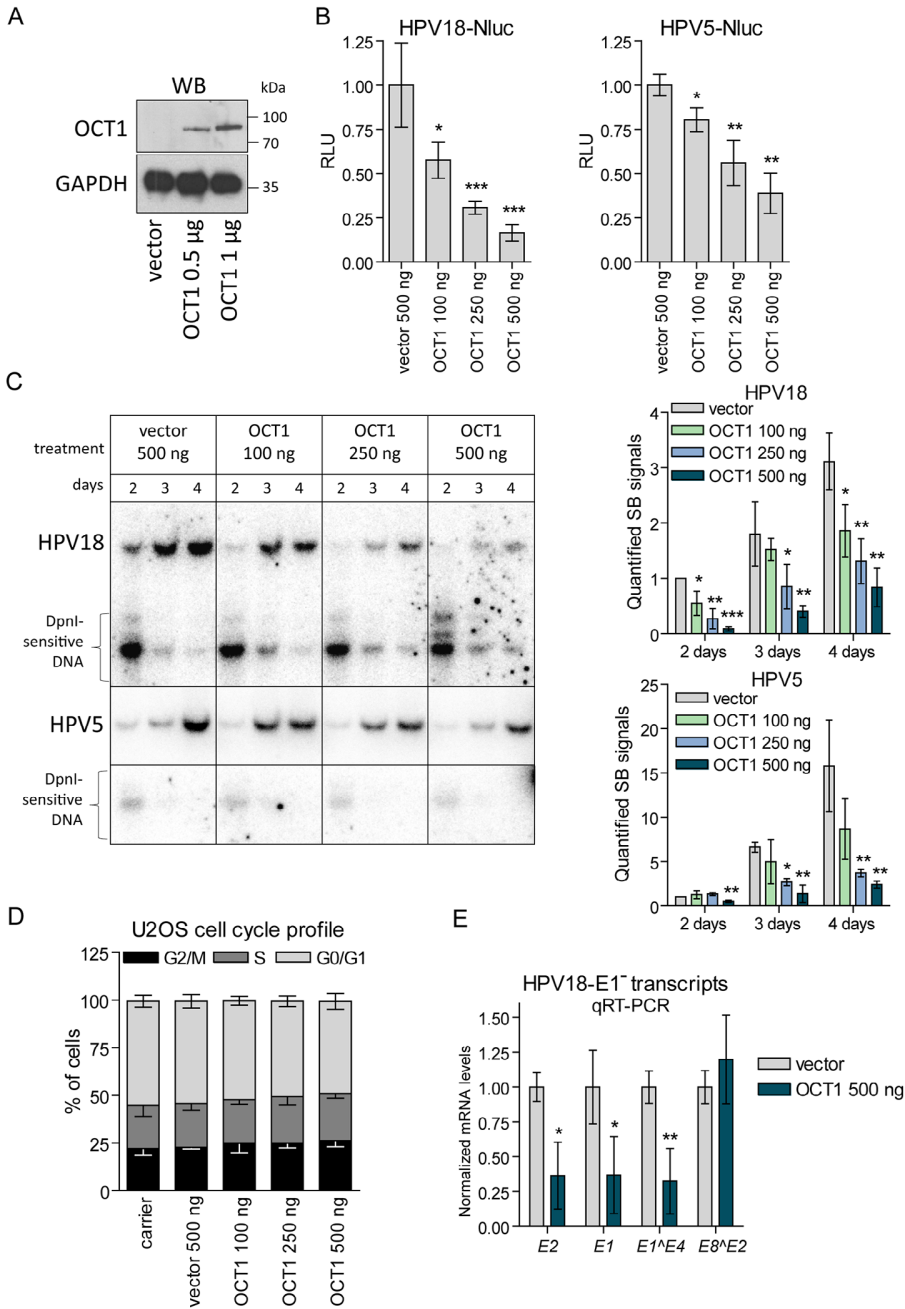
OCT1 Down-regulates Replication of HPV18 and HPV5 Genomes (**A**) OCT1 was overexpressed in U2OS cells. WCEs were analysed using immunoblotting and anti-OCT1 antibody 2 days post-transfection. GAPDH is shown as a loading control. (**B**) U2OS cells were cotransfected with either HPV18-Nluc or HPV5-Nluc genomes and the indicated amounts of the OCT1 expression construct. Nluc activity was measured in triplicate using the Live Cells Assay and normalized to viability values (MTS assay) 3 days post-transfection. Normalized Nluc activity in the control samples (cotransfected with an empty vector) was set as 1, and data from the OCT1-treated samples are expressed relative to the control. (**C**) The OCT1 expression vector at the amounts indicated was electroporated together with either HPV5 or HPV18 genomes per million U2OS cells, which were split into separate wells for acquiring multiple timepoints (shown by days). Total DNA was treated with DpnI and BglI or SacI restriction endonucleases to linearize the HPV18 and HPV5 genomes, respectively. The replication rate of HPV genomes was analysed using SB (left panel), and the obtained signals were quantified (right panels). (**D**) U2OS cells were transfected with the HPV18 genome together with carrier DNA, an empty vector or various quantities of the OCT1 expression vector. Cell cycle profiles were analysed using propidium iodide by flow cytometry 3 days post-transfection. (**E**) U2OS cells were transfected with the HPV18-E1^-^ genome and OCT1 expression construct. Total RNA was isolated 3 days post-transfection. Viral gene mRNA expression levels were analysed in triplicate using qPCR, normalized to *GAPDH* expression levels, and set as 1 in the samples cotransfected with an empty vector. Data from the OCT1-treated samples are presented relative to 1. All panels: data are presented as average means ± SDs of 3 independent experiments (* - *p* < 0.05; ** - *p* < 0.01; *** - *p* < 0.001)

To investigate the impact of POU-HD TFs on HPV genome replication, we initiated studies using U2OS cells, known for their efficient support of transfected HPV genome replication [25]. OCT1, a representative member of the POU-HD TF family expressed in U2OS cells, was selected for initial investigations. Western blot (WB) analysis confirmed the expression of exogenous OCT1 in transfected cells (Fig. 2A).

U2OS cells were transfected with modified HR alpha HPV18-Nluc or beta HPV5-Nluc genomes, along with increasing amounts of the OCT1 expression vector. These genomes enabled the expression of the Nluc gene, with activity correlates to genome copy numbers. Nluc activity, measured 3 days post-transfection and normalized to alkaline phosphatase activity, was set as 1 in control cells cotransfected with the HPV genome and an empty vector (Fig. 2B). Overexpression of OCT1 led to a dose-dependent reduction in Nluc activity.

To validate these findings, we utilized wild-type (WT) HPV5 and HPV18 genomes cotransfected with increasing amounts of the OCT1 expression vector to assess viral genome replication through Southern blot (SB) analysis. The results demonstrated a concentration-dependent inhibition of HPV5 and HPV18 genome replication upon OCT1 overexpression (Fig. 2C). Quantitative analysis from three independent experiments confirmed the statistically significant repression of viral genome replication by OCT1 (Fig. 2C, bottom panels).

Considering the potential influence of OCT1 on the cell cycle, we examined the cell cycle profile of U2OS cells transfected with increasing amounts of the OCT1 expression vector. Notably, even at the highest concentrations, OCT1 did not significantly perturb the U2OS cell cycle (Fig. 2D). Additionally, a slight, gradual shift of cells from G0/G1 towards G2/M and S phases was observed in an OCT1 concentration-dependent manner. At the highest OCT1 concentration, approximately 5% fewer cells were in G0/G1, creating more favourable conditions for HPV genome replication [26]. These observations indicate that the inhibitory effect of OCT1 on HPV5 and HPV18 replication is independent of the cell cycle.

To delve into the mechanism of OCT1-mediated downregulation of HPV replication, we analysed HPV18 transcription in the presence of overexpressed OCT1. To minimize contamination with replicated HPV18 DNA, we cotransfected U2OS cells with the replication-deficient HPV18-E1^−^ genome and either the OCT1-encoding construct or an empty vector as a control. qPCR analysis, performed 2 days post-transfection, revealed that overexpressed OCT1 significantly downregulated the expression of the viral transcripts *E1*, *E1^E4*, or *E2* initiated from the promoters P102, P520, or P811, while having no effect on the *E8^E2* transcript expressed from the promoter P1193 (Fig. 2E). This finding indicates that OCT1 modulates HPV18 transcription, providing deeper insights into the molecular mechanism of its inhibitory effect on HPV replication.

### Several members of the POU-HD family of TFs inhibit replication of the HPV5, HPV11, and HPV18 genomes

**Fig. 3:**
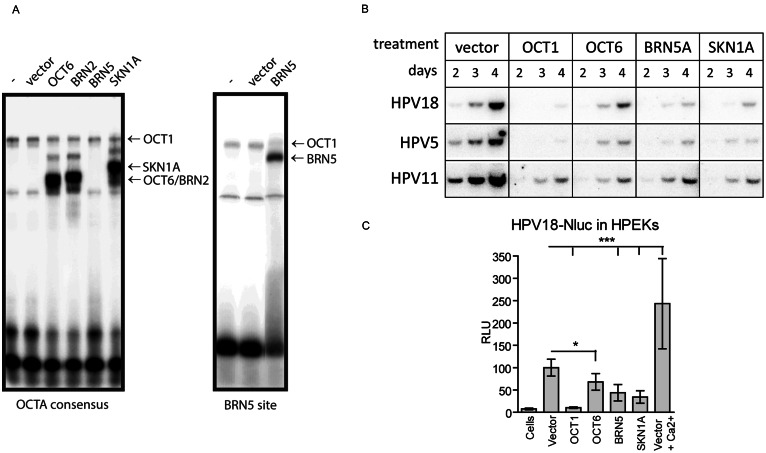
POU-HD TFs Inhibit Replication of HPV18, HPV5, and HPV11 Genomes (**A**) U2OS cells were transfected with the appropriate expression vector and incubated for 2 days, and WCEs were prepared. Equal amounts of the WCEs were incubated with 32-P-labelled double-strand oligonucleotides for 30 min and resolved on 4% PAAG. The relative mobility of different protein-DNA complexes was deduced from published scientific literature. Right panel: the image has been cropped for clarity of the presentation. (**B**) U2OS cells were cotransfected with viral genomes and either an empty vector or the indicated expression constructs using 500 ng of the plasmids per 10^6^ cells. Cells were split after transfection to acquire different time points and incubated for 2, 3, and 4 days. Total DNA was purified, treated with the restriction enzymes DpnI and BglI, SacI or HindIII to linearize the HPV18, HPV5, and HPV11 genomes, respectively, and analysed using SB. (**C**) HPEKs were transfected with the HPV18-Nluc genome and the indicated expression constructs or an empty vector. The days after transfection, HPEKs were treated with 1.5 mM CaCl_2_ to induce differentiation. Nluc activity was measured 3 days post-transfection, normalized to alkaline phosphatase values, and set as 100% in the control samples transfected with an empty vector. Data from other samples are presented as a percentage ± SD of the control (*n* = 3, * - *p* < 0.05, *** - *p* < 0.001)

Next, we analysed the effects mediated by other POU-HD family TFs on the replication of the HPV5, HPV11, and HPV18 genomes. Given that different POU-HD TFs bind to similar consensus sequences on DNA in vitro, we overexpressed OCT1, OCT6, BRN5, SKN1A, and BRN2 in U2OS cells and verified their ability to bind specific DNA elements by EMSAs, showing that all used constructs expressed the respective POU-HD protein that was able to bind DNA (Fig. 3A). We used the OCTA consensus sequence (ATTTGCAT) to detect protein-DNA complexes for OCT1, OCT6, SKN1A, and BRN2 and the nonoctamer sequence element derived from the corticotrophin-releasing hormone gene promoter (GCATAAATAAT) to detect BRN5.

Next, we cotransfected U2OS cells with the HPV genomes and 500 ng of OCT1, OCT6, BRN5, and SKN1A expression constructs or an empty vector. Since BRN2 and BRN3A are not expressed in HPV host cells, they were excluded from further studies. Total DNA was isolated 2, 3, and 4 days post-transfection, and the amounts of replicated linearized viral genomes were analysed using SB (Fig. 3B). Our analysis showed that OCT6, BRN5, and SKN1A, similar to OCT1, inhibited replication of the HPV5, HPV11, and HPV18 genomes in U2OS cells.

To confirm the obtained results in HPEKs, we analysed the effect of POU-HD TFs on replication of the HPV18-Nluc genome chosen as a prototype of an oncogenic virus type. HPEKs were cotransfected with the HPV18-Nluc genome and the expression vectors coding for OCT1, OCT6, BRN5, SKN1A or an empty vector. Additionally, HPEKs were treated with Ca^2+^ to analyse the HPV18-Nluc relative copy numbers in differentiating cells. Nluc activity was measured 3 days post-transfection and normalized to alkaline phosphatase values (Fig. 3C). Our analysis revealed that overexpression of OCT1, OCT6, BRN5, and SKN1A, or POU-HD TFs expressed in HPEKs, inhibited HPV18-Nluc replication. OCT1 elicited the strongest inhibitory effect, and the relative Nluc activity in these cells was similar to that in the nontransfected cells. In the presence of BRN5 and SKN1A, HPV18-Nluc copy numbers decreased by approximately 60%, and OCT6 inhibited Nluc activity by approximately 30%. In contrast, upregulation of Nluc activity was detected in response to Ca^2+^, indicating an increase in HPV18-Nluc genome copy numbers in differentiating cells. Taken together, these data show that several members of the POU-HD TF family are able to inhibit replication of the HPV5, HPV11, and HPV18 genomes.

### Knockdown of endogenous *OCT1* expression results in an increase in the replication efficiency of the HPV18 genome

Our data demonstrate the expression of several POU-HD TFs, namely, *OCT1*, *BRN5A*, *SKN1A*, and *OCT6*, in HPEKs and U2OS cells. When overexpressed, these proteins inhibit the replication of different HPV genomes. However, overexpression of a TF might induce nonphysiological effects due to excessively high intracellular levels of the protein. Conversely, the ability of POU-HD TFs to bind to an octamer ATGCTAAT-like consensus sequence may lead to functional redundancy, complicating the effects of individual POU-HD TF gene silencing. As simultaneous RNA interference (RNAi) for all POU-HD TFs is technically impossible, we strengthened our findings by assessing the impact of *OCT1* RNAi on the replication of the HPV18 genome in U2OS cells, considering the background of endogenous BRN5A and SKN1A expression.

**Fig. 4:**
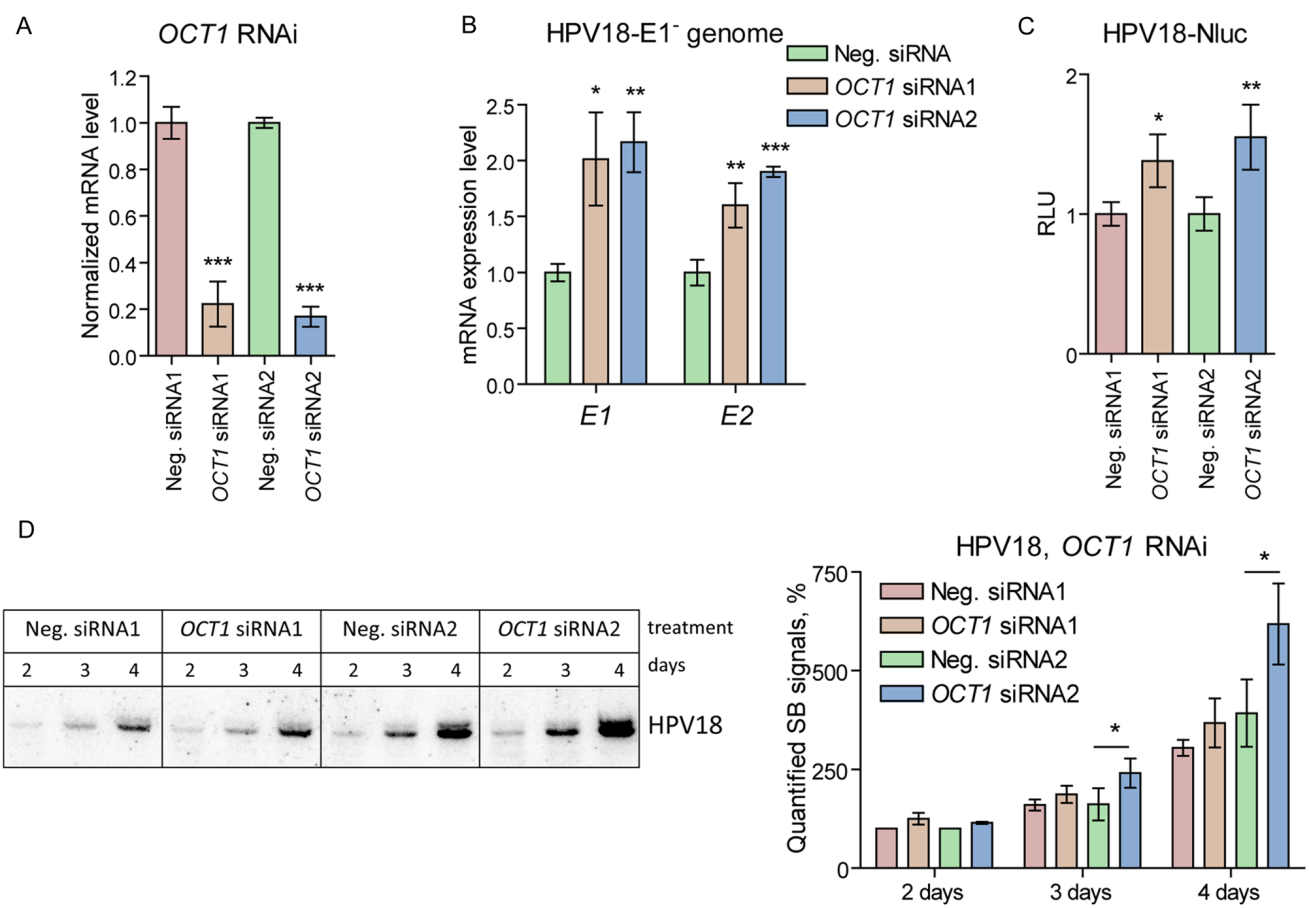
*OCT1* RNAi Results in the Upregulation of HPV18 Genome Replication (**A**) U2OS cells were transfected with 2 different scrambled siRNAs (Neg. siRNAs) or *OCT1*-specific siRNAs. *OCT1* mRNA expression levels were measured using qPCR 2 days post-transfection, normalized to *GAPDH* mRNA expression levels and set as 1 in the samples transfected with Neg. siRNAs. Data from other samples were calculated relative to the respective control. (**B**) U2OS cells were cotransfected with the HPV18-E1^-^ genome and scrambled or *OCT1*-specific siRNAs. *E1* and *E2* mRNA expression levels were measured using qPCR 3 days post-transfection, normalized to *GAPDH* expression levels and set as 1 in the control sample transfected with Neg. siRNA. Data from other samples are presented relative to the control. (**C**, **D**) U2OS cells were cotransfected either with HPV18-Nluc or HPV18 genomes and scrambled or *OCT1*-specific siRNAs. Viral genome copy numbers were analysed using either the Nluc assay (**B**) or SB (**C**). Prior to SB, total DNA was treated with the BglI and DpnI restriction enzymes. All panels: data are shown as an average mean ± SD, * - *p* < 0.05, ** - *p* < 0.01, *** - *p* < 0.001, *n* = 3

*OCT1* expression was interfered with using two different siRNAs, and their efficiency was measured 2 days post-transfection through qPCR. SiRNA1 and siRNA2 reduced *OCT1* mRNA levels by 79% and 85%, respectively (Fig. 4A). To demonstrate the role of OCT1 in viral early gene transcription, we cotransfected U2OS cells with the replication-deficient HPV18-E1^−^ genome and either *OCT1*-specific or scrambled siRNAs. *E1* and *E2* mRNA expression levels were measured 3 days post-transfection using qPCR and normalized to *GAPDH* expression levels (Fig. 4B). *OCT1* RNAi led to up-regulation of *E1* and *E2* expression.

Subsequently, we cotransfected U2OS cells with *OCT1* siRNAs and either the HPV18-Nluc or HPV18 genomes. Replication was assessed using the Nluc assay or SB at 2, 3, and 4 days post-transfection (Fig. 4C and D, respectively). In both cases, *OCT1* RNAi led to a slight increase in viral genome copy numbers, although this change was mostly statistically nonsignificant in the case of the quantified SB signals.

### Inhibition of HPV18 replication depends on the DNA binding activity of Oct6

**Fig. 5:**
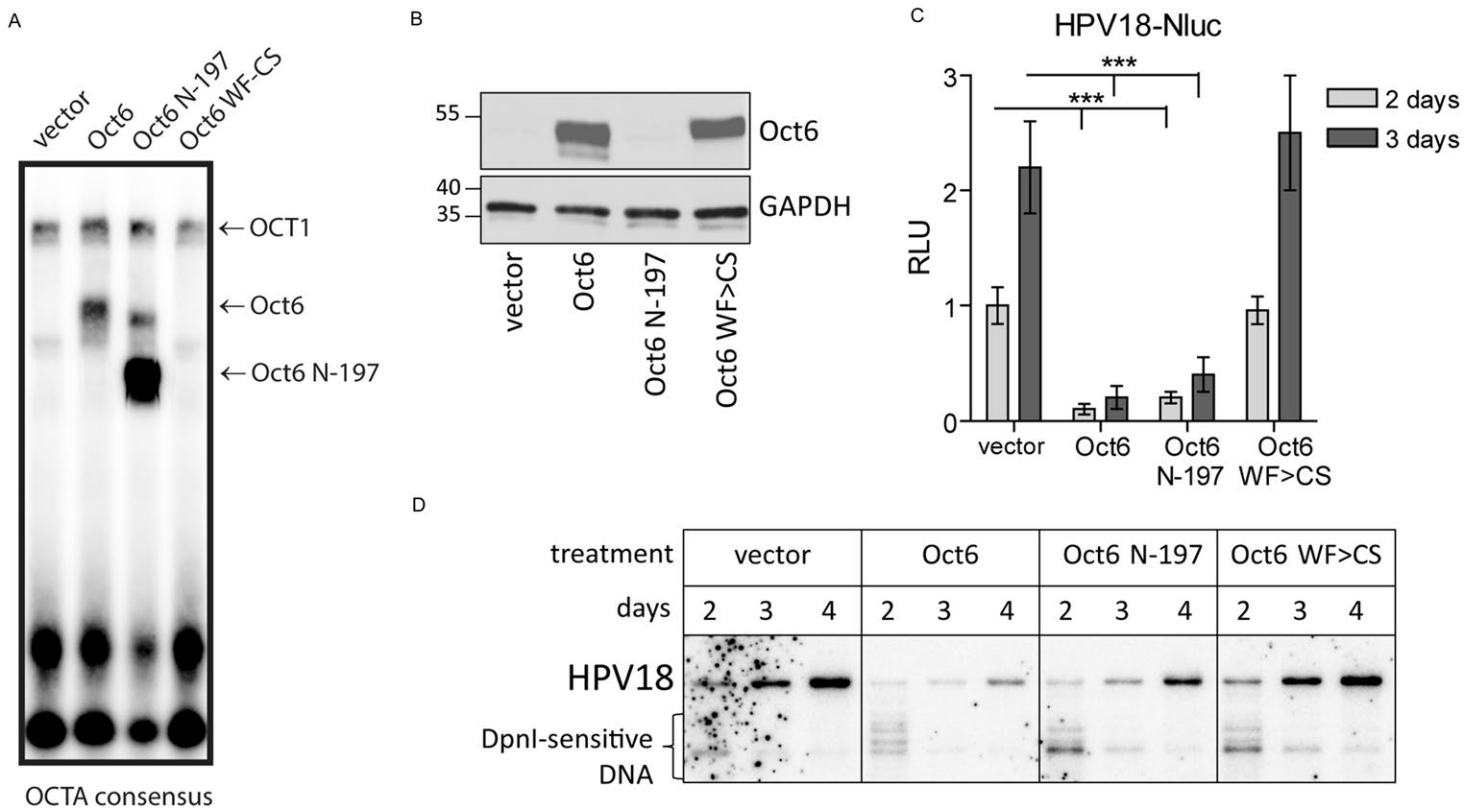
DNA Binding is Required for the Oct6-Mediated Inhibition of HPV18 Replication (**A**) U2OS cells were transfected with the appropriate expression vectors. WCEs were prepared 2 days after transfection. Equal amounts of WCEs were incubated with 32-P-labelled double-strand oligonucleotides for 30 min and resolved on native 4% PAAG. (**B**) WT Oct6, Oct6-N-197, and Oct6-WF > CS proteins were overexpressed in U2OS cells. WCEs were analysed using immunoblotting and anti-Oct6 or GAPDH antibodies 2 days post-transfection. (**C**) U2OS cells were cotransfected with the HPV18-Nluc genome and expression constructs encoding either WT Oct6 or its mutants, Oct6-N197 and Oct6-WF > CS. An empty vector was used as a control. Nluc activity was measured 2 and 3 days post-transfection and normalized to that of AP. Normalized Nluc activity in the control cells was set as 1, and data of other samples were calculated relative to the control. Data are shown as an average mean ± SD, *n* = 3, ***- *p* < 0.001. (**D**) U2OS cells were cotransfected with the HPV18 genome and either an empty vector or Oct6 expression constructs. Total DNA was isolated 2, 3, and 4 days after transfection, treated with the BglI and DpnI restriction enzymes to linearize the HPV18 genome and digest the input DNA, respectively, and analysed using SB

Next, we investigated, whether the negative effect of POU-HD TFs on HPV18 replication depended on the DNA binding and/or transcriptional regulation ability of the proteins. To address this, we utilized Oct6 and its mutants, Oct6-N197 and Oct6-WF > CS, as an experimental model. The Oct6-N197 protein lacks the first 197 amino acids; this protein retains the ability to specifically bind DNA but is deficient in the transactivation function. The Oct6-WF > CS mutant contains point mutations in the DNA binding domain, disrupting the specific association of the protein with DNA. EMSA analysis confirmed the presence of both WT Oct6 and Oct6-N197 in transfected cells, while Oct6-WF > CS did not bind DNA (Fig. 5A). Similar levels of the overexpressed WT Oct6 and Oct6-WF > CS proteins were detected using immunoblotting and anti-Oct6 antibody that was generated against the Oct6 N-terminal peptide and does not recognize the Oct6-N197 protein (Fig. 5B).

Cotransfection of WT or mutant Oct6 expression vectors with the HPV18-Nluc genome into U2OS cells, followed by analysis of Nluc activity at 2 and 3 days post-transfection, revealed that both WT Oct6 and Oct6-N197 mutants were able to inhibit HPV18 replication by approximately 80%, whereas the Oct6-WF > CS mutant did not (Fig. 5C). This result was confirmed by cotransfection of expression vectors encoding WT and mutant Oct6 proteins with the WT HPV18 genome, followed by direct analysis of viral genome replication using SB (Fig. 5D). These data indicate that Oct6-mediated inhibition of HPV18 replication depends solely on its DNA binding activity, while the N-terminal domain of Oct6 is dispensable for the decrease in HPV18 genome replication.

## Discussion

### Expression of POU factors in keratinocytes

Several members of the POU-HD family of TFs play crucial roles in regulating cellular processes in keratinocytes. Prior studies have established the expression of OCT1, OCT6, and SKN1A in neonatal human keratinocytes [27, 28]. Additionally, BRN5 expression has been identified in rat skin [29]. BRN2 is expressed in differentiated keratinocytes, while BRN3A is associated with expression in CIN lesions of the cervix [30, 31]. Mouse knockout studies have highlighted the functional importance of POU factors in skin, particularly revealing a severe hyperplastic skin phenotype upon simultaneous inactivation of OCT6 and SKN1A [32]. Our study extends this understanding by identifying *BRN5* expression in primary keratinocytes, in addition to the previously known expression of *OCT1*, *OCT6*, and *SKN1A*. We did not detect expressions of *BRN2* or *BRN3A*. Notably, the absence of *BRN3A* expression aligns with the in vivo findings, reinforcing that *BRN3A* is expressed exclusively in cancerous lesions of the cervix [33]. The disparity in *BRN2* results between our study and prior data may stem from differences in experimental setups. In Shi et al.‘s work, *BRN2* expression occurred in keratinocytes after 14 days of differentiation with elevated Ca levels [30]. In contrast, our protocol employed elevated Ca^2+^ for up to 3 days in keratinocyte differentiation.

### POU-HD factors inhibit HPV genome replication

Previous research has indicated a positive effect of SKN1A overexpression on the replication of HPV16 ori-bearing plasmids [15]. Additionally, luciferase assays have suggested a favourable impact of POU-HD factors, including SKN1A, OCT6, and BRN3A, on the activation of HPV early promoters [16–18]. In our study, we demonstrate that overexpression of POU-HD factors — OCT1, OCT6, SKN1A, and BRN5, — results in the inhibition of HPV5, HPV11, and HPV18 genome replication in the U2OS cells and primary keratinocytes, which are both permissive for HPV replication.

Discrepancies with prior data are explicable; earlier replication assays utilized an HPV16 ori-containing plasmid coexpressed with HPV16 E1 and E2 and SKN1A expression plasmids. This setup led to high expression levels of HPV replication proteins from heterologous promoters, lacking physiological relevance. Moreover, these assays were conducted in HEK293 cells, where HPV genomes are incapable of replication. Our results, supported by RNAi experiments showing a slight increase in HPV18 replication upon OCT1 inhibition in U2OS cells, further confirm that POU-HD factors indeed inhibit HPV replication.

Our findings suggest that the primary mechanism of POU-HD factor-mediated inhibition of HPV replication involves modulation of HPV early promoters. Moreover, we demonstrate that the inhibitory effect of at least OCT6 is contingent upon its binding to DNA, as the OCT6 mutant incapable of binding DNA fails to inhibit HPV18 replication. Intriguingly, the activation domain of OCT6 was dispensable for repressing HPV18 replication. This finding implies that the binding of OCT6 to HPV18 may induce a steric hindrance effect, disrupting the formation of protein complexes needed for efficient transcription from the viral genome.

Our data indicate a reduction in *E2* and *E1* transcript levels in response to the OCT1 overexpression, and an up-regulation of *E1* and *E2* expression in response to the *OCT1* RNAi. *E2* transcripts arise from HPV18 promoters P102 and P811, while *E1* transcripts exclusively arise from P108 [34, 35]. Furthermore, OCT1 overexpression lowers *E1/E4* transcript levels, originating solely from P811. Notably, OCT1 exerts no effect on the HPV18 promoter P1193, which drives the expression of *E8/E2*, a viral negative regulator of replication [24]. These data suggest that presence of OCT1 results in decreased levels of the HPV replication proteins E1 and E2, while the levels of the viral negative regulator of replication, E8/E2, remain unchanged. In the context of the viral genome, a shift towards E8^E2 results in decreased replication and lower copy numbers of the genome.

The HPV18 promoter P102 partially overlaps with the viral ori, harbouring POU-HD binding sites predicted to partially overlap with the binding site for the viral initiator E1 and the cellular TF SP1. Consequently, POU-HD factor binding to these sites in the viral ori could negatively impact both viral replication initiation and transcription from P102.

HPV18 P811, initially described as a late promoter, has been shown to be active in U2OS cells transfected with HPV18 genomes [34, 35]. Furthermore, viral mRNAs from this promoter are polyadenylated at an early polyA signal, indicating its activity in the early stages of viral infection. HPV18 P811 contains a cis-acting repressor element (AAGTATGCA) that interacts with hnRNP D0B and hnRNP A/B factors [36]. This sequence resembles the POU-HD binding site, and various TF DNA binding prediction programs suggest the binding of OCT1 to this site. Therefore, the repressive effect of POU-HD TFs on HPV18 P811 may occur through the shared DNA element with hnRNP factors.

### The role of POU-HD TFs in HPV genome replication dynamics during keratinocyte differentiation

We have demonstrated that Ca^2+^-induced differentiation of HPEKs results in the upregulation of endogenous *OCT6* and *SKN1A* expression. These TFs inhibit HPV DNA replication in overexpression studies. These findings suggest that elevated expression of OCT6 and SKN1A should either decrease or restrict the increase in HPV genome copy numbers in differentiating keratinocytes. In reality, however, the copy numbers of HPV genomes increase approximately 2-fold upon Ca^2+^-induced differentiation of HPEKs. One possibility to explain this phenomenon is that the elevated levels of OCT6 and SKN1A expression are not sufficient to exert an extensive negative effect on HPV genome copy numbers. For instance, if the regulation of HPV replication occurs through competitive binding of the negative (e.g. POU-HD TFs) and the positive regulators (replication initiation factors, e.g. E1 and/or E2), the balance is still strongly on the side of the positive regulators in the analysed HPEKs. Also, it is plausible to speculate that POU-HD TFs still restrict the increase in HPV copy numbers in differentiating keratinocytes, however, to an unknown extent. Additional studies are needed to explore the effect of POU-HD TFs RNAi on HPV genome copy numbers in these experimental settings.

Another possibility is that the increase in HPV copy number during Ca^2+^-induced differentiation of HPEKs is not due to an increase in the initiation of HPV replication per se, but rather more efficient elongation and/or partitioning of the replicated episomes. It has been shown that HPV replication is inefficient in the sense that a lot of semi-replicated viral genome molecules are still detectable in the G2/M phase of the cell cycle [37]. Differentiation of the cells might result in a more favourable environment to complete viral genome replication. Hypothetically, as POU-HD TFs control the initiation of HPV replication, they may have no role in these processes.

Our data show that the main mechanism of action of the POU-HD TFs on HPV replication is the negative regulation of the HPV early promoters and/or replication ori activity. Based on the data presented in this article, it is plausible to speculate that one possible inhibitory effect of POU-HD proteins could be the suppression of over-replication of the viral genomes in both the initial and latent stages of the viral infection. This mechanism may help the virus to escape the activity of the host’s immune system and to prevent abortive infection and cell death.

In fact, there is very little understanding of how the latent stage of the infection is initiated and why the replication of the viral genome is slowed down, so that it becomes a once-per-cell-cycle type. One possibility is that as the expression of POU-HD factors is elevated during the differentiation of the keratinocytes, this elevated expression contributes to the negative regulation of the viral genome replication and initiates stable replication of the viral genomes that occurs in the intermediate levels of the infected stratified epithelium. Finally, the biological consequences of the POU-HD TF-mediated effect may involve restricting viral genome replication in the upper, more differentiated layers of the epithelium before the initiation of virion packaging.

## Conclusions

Our data strongly suggest that the POU-HD TFs OCT1, OCT6, BRN5A, and SKN1A are expressed in HPV host cells and inhibit the replication of HPV genomes by binding to viral DNA and modulating viral transcription. Our study emphasizes the intricate regulatory roles of POU-HD factors in the HPV replication.

### Electronic supplementary material

Below is the link to the electronic supplementary material.

Supplementary Material 1

Supplementary Material 2

## Data Availability

No datasets were generated or analysed during the current study.
