# Supplementary material for: The POU-HD TFs impede the replication efficiency of several human papillomavirus genomes
Source: Virol J. 2024 Mar 5;21:54. doi: 10.1186/s12985-024-02334-w (PMC10916165; doi:10.1186/s12985-024-02334-w)
Supplement: Supplementary file 1 — Supplementary Material 1 [file 12985_2024_2334_MOESM1_ESM.docx]

Supplementary Table S1. Oligonucleotides used in the present study.

| *GAPDH* | sense | CTCTCTGCTCCTCCTGTTCGAC |
| --- | --- | --- |
|  | antisense | TGAGCGATGTGGCTCGGCT |
| HPV18 *E1* | sense | CATTTACCAGCCCGACGAG |
|  | antisense | AAACCAGCCGTTACAACCCG |
| HPV18 *E1* | sense | GATAGTGGCTATGGCTGTTC |
|  | antisense | GCTGTTGTTGCCCTCTGTG |
| HPV18 *E1^E4* | sense | CATTTACCAGCCCGACGAG |
|  | antisense | GACGTCTGGCCGTAGGTCTTTGC |
| HPV18 *E8^E2* | sense | GATAGTGGCTATGGCTGTTC |
|  | antisense | GACGTCTGGCCGTAGGTCTTTGC |
| HPV18 *E8^E2* | sense | CTGAAGTGGAAGCAACACAG |
|  | antisense | GACGTCTGGCCGTAGGTCTTTGC |
| HPV18 *E2* | sense | AGTACCAGTGACGACACGGT |
|  | antisense | GACGTCTGGCCGTAGGTCTTTGC |
| *IVL* | sense | TTCCTCCTCCAGTCAATACC |
|  | antisense | CTCAGGCAGTCCCTTTACAG |
| *IVL* | sense | TCCTCCAGTCAATACCCATCAG |
|  | antisense | GCAGTCATGTGCTTTTCCTCTTG |
| *K10* | sense | TTGCTGAACAAAACCGCAAAG |
|  | antisense | GCCAGTTGGGACTGTAGTTCT |
| *K10* | sense | GGGCTCTGGAAGAATCAAAC |
|  | antisense | CTGGCATTGTCGATCTGAAG |
| *OCT6* | sense | CATGTGCAAGCTCAAGCC |
|  | antisense | CGATCTTGTCCAGGTTGGT |
| *OCT1* | sense | GGCTAAATGATGCAGAGAACC |
|  | antisense | TTTCTTCCTCCTACGGCTC |
| *SKN1A* | sense | CCCATCAAACCACCTGTC |
|  | antisense | AGGATGACGTTACTGTTCCA |
| *SKN1A* | sense | GAATGATGCAGAGTCCTCTCC |
|  | antisense | GTTATCTTGAAACCTCTTCTCCAG |
| *BRN3A* | sense | AGCCTCACTTTGCCATGC |
|  | antisense | GAAGAGGTTGCTCTGCAGC |
| *BRN3A* | sense | GAACAGCAAGCAGCCTCACTT |
|  | antisense | CAGCAGCGTCTCGTCCAG |
| *BRN2* | sense | CTGGGCACCCTGTATGGCAACG |
|  | antisense | GGCTTCAGCTTGCACATG |
| *BRN2* | sense | GCGGATCAAACTGGGATTTA |
|  | antisense | AAAGGCTTCAGCTTGCACAT |
| *BRN5* | sense | GCTCAGGGACAGGTTATTGG |
|  | antisense | CTGGGCGTTCAACAACAG |
| *BRN5* | sense | GTCCCTCACTACAGCTCCA |
|  | antisense | GTTCCAATAACCTGTCCCTGAG |
