# Supplementary figures and images for: The POU-HD TFs impede the replication efficiency of several human papillomavirus genomes
Source: Virol J. 2024 Mar 5;21:54. doi: 10.1186/s12985-024-02334-w (PMC10916165; doi:10.1186/s12985-024-02334-w)

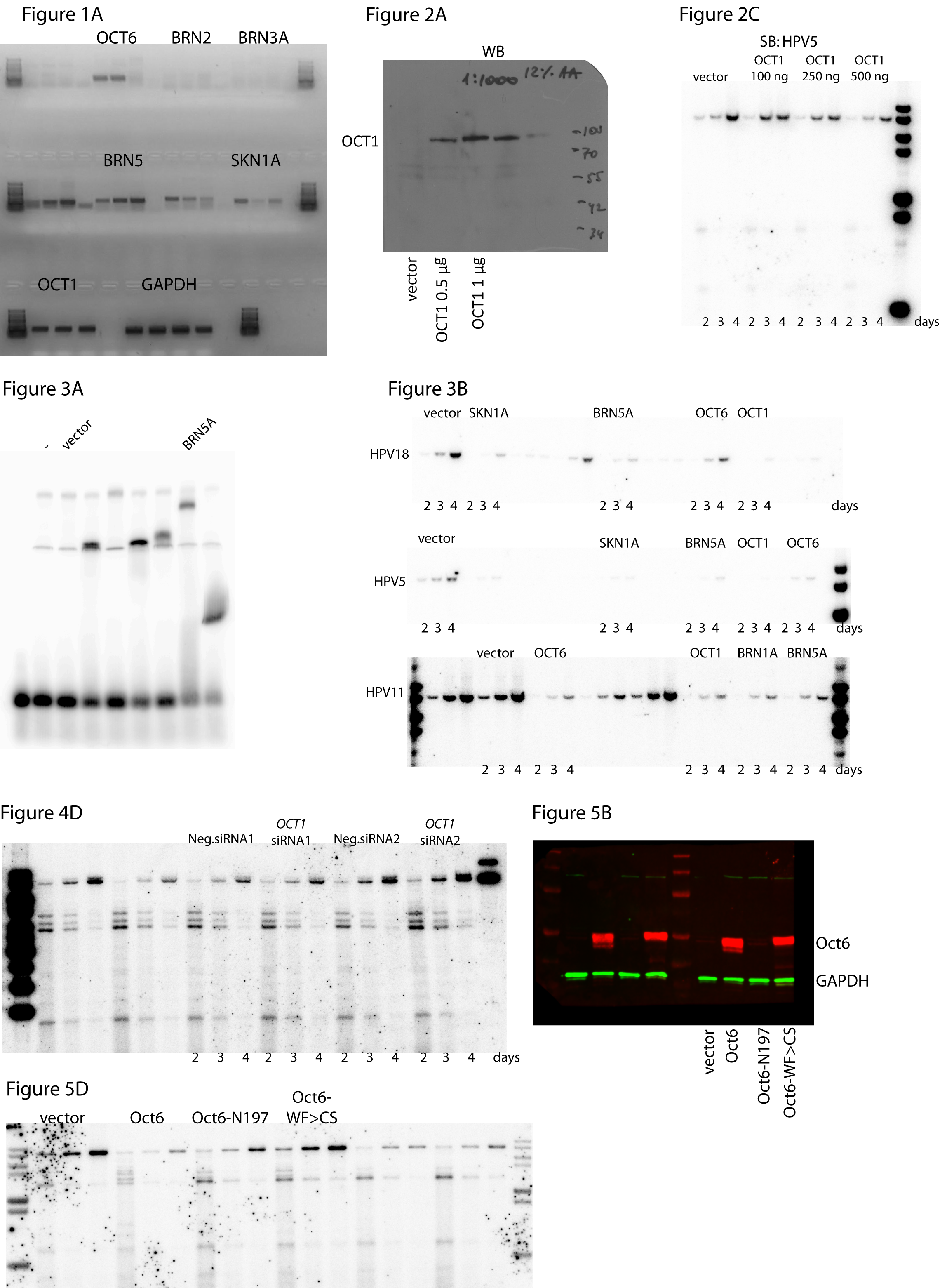

Supplement: Supplementary file 2 — Supplementary Material 2 [file 12985_2024_2334_MOESM2_ESM.tif]
